# Vestibular evoked myogenic potential may predict the hearing recovery in patients with unilateral idiopathic sudden sensorineural hearing loss

**DOI:** 10.3389/fneur.2022.1017608

**Published:** 2022-11-02

**Authors:** Min Liang, Hui Wu, Jianyong Chen, Qin Zhang, Shuna Li, Guiliang Zheng, Jingchun He, Xiangping Chen, Maoli Duan, Jun Yang, Yulian Jin

**Affiliations:** ^1^Department of Otolaryngology-Head and Neck Surgery, Xinhua Hospital, Shanghai Jiaotong University School of Medicine, Shanghai, China; ^2^Ear Institute, Shanghai Jiao Tong University School of Medicine, Shanghai, China; ^3^Shanghai Key Laboratory of Translational Medicine on Ear and Nose Disease, Shanghai, China; ^4^Department of Otolaryngology-Head and Neck Surgery, Karolinska University Hospital, Karolinska Institute, Stockholm, Sweden; ^5^Department of Clinical Science, Intervention and Technology, Karolinska Institute, Stockholm, Sweden

**Keywords:** unilateral idiopathic sudden sensorineural hearing loss, vestibular function, vestibular evoked myogenic potential, caloric test, otolith organ, sacculus, utriculus

## Abstract

**Objective:**

This study investigates the association between vestibular function and prognosis in patients with unilateral idiopathic sudden sensorineural hearing loss (UISSNHL).

**Design:**

A retrospective analysis of 64 patients with UISSNHL was performed. Pure tone audiometry and vestibular function tests for otoliths and semicircular canals were performed to assess the influence of vestibular functional status on the outcome of patients with UISSNHL.

**Results:**

Patients with abnormal cervical vestibular evoked myogenic potential (cVEMP) or ocular vestibular evoked myogenic potential (oVEMP) responded less favorably to treatment. In the ineffective group, cVEMP was normal in four patients (6.3%) and oVEMPs in three (4.7%). Meanwhile, cVEMP was abnormal in 32 patients (50.0%) and oVEMP in 33 (51.6%). Better hearing recovery occurred in those with normal cVEMP (33.76 ± 15.07 dB HL improvement) or oVEMP (32.55 ± 19.56 dB HL improvement), but this was not the case in those with normal caloric tests. Patients with abnormalities in both cVEMP and oVEMP were less responsive to treatment and had worse hearing recovery than those with normal results in only one of the two tests.

**Conclusion:**

Abnormal oVEMP and/or cVEMP results indicate poor auditory outcomes in patients with UISSNHL. Patients with impaired otolith organ function are likely to have a larger and more severe pathological change in their inner ear.

## Introduction

Sudden sensorineural hearing loss (SSNHL) is a serious, rapid-onset inner ear disease without known etiology. It is defined as a sensorineural hearing loss of at least 30 dB over at least three connected/consecutive frequencies (1, 2) occurring within 72 h (3). Various postulated etiological theories have been proposed in the literature, including viral infection, vascular embolism, and metabolic abnormalities (4–7). Nearly 40%−55% of patients with SSNHL show vestibular symptoms such as dizziness and instability, which can be delayed or occur at the same time with sudden hearing loss (8–10). This suggests that cochlear impairment and vestibular dysfunction can accompany each other (8, 9, 11–13).

The cochlear and vestibular embryos are homologous. The otic vesicles are derived from the otic placodes situated on either side of the embryonic hindbrain and differentiate into superior vestibular and inferior cochlear parts (14, 15). As a disease of unknown etiology, SSNHL can cause damage to both cochlear and vestibular organs, which share a common origin in terms of cochlear and vestibular arteries (16). A poor prognosis was reported in SSNHL patients with vertigo (3, 4, 17–20). However, the underlying vertigo assessment of peripheral vestibular organs has not yet been well described.

Various vestibular function tests may assist in mapping the affected area of vestibulopathy and provide information that improves the prediction of hearing outcomes. Vestibular evoked myogenic potential (VEMP), including cervical vestibular evoked myogenic potential (cVEMP) and ocular vestibular evoked myogenic potential (oVEMP), is recorded on the surface of the skeletal muscle under tension evoked by strong acoustic stimulation on vestibular terminal sensors. They reflect the functions of the sacculus and utriculus, respectively. The caloric test is clinically used to examine the horizontal semicircular canal and the supra-vestibular nerve pathways. When combined with audiometric tests, the vestibular function test battery, including cVEMP, oVEMP, and caloric test, can provide a more accurate and comprehensive assessment of the cochlear and vestibular system to check the function of almost the entire inner ear (cochlea, sacculus, utriculus, and horizontal semicircular canal).

To date, the involvement of vestibular organs in SSNHL remains controversial. Moreover, it is still unknown whether detecting normal or abnormal VEMP responses is useful in determining a patient's prognosis. This study aims to analyze the relationship between the aforementioned vestibular function as part of a complete neurotological evaluation and auditory outcome in patients with UISSNHL and to determine the possible predictive significance of vestibular function in the participating patients.

## Materials and methods

### Patients

In total, 64 patients with UISSNHL were recruited from the Department of Otolaryngology-Head and Neck Surgery, Xinhua Hospital, affiliated with the Shanghai Jiaotong University School of Medicine, from May 2017 to July 2021. This included 38 men and 26 women aged between 18 and 87 years (average of 53.75 ± 17.00 years), with moderate deafness in four ears, moderate to severe deafness in 15 ears, severe deafness in six ears, profound deafness in 13 ears, and total deafness in 26 ears. Of the patients, 28 had hearing loss in the left ear and 36 in the right ear. Vertigo, dizziness, or unsteadiness appeared in 35 patients. The diagnosis of UISSNHL followed the American Academy of Otolaryngology-Head and Neck Surgery Foundation's (AAOHNSF) “Clinical Practice Guideline: Sudden Hearing Loss” (2). All human procedures were approved by the institutional review board in Xinhua Hospital, affiliated with the Shanghai Jiaotong University School of Medicine. All participants provided verbal informed consent.

The inclusion criteria were as follows: ① age ≥ 18 years; ② an unknown cause; ③ moderate to total hearing loss; ④ initiation of treatment within 30 days after onset; and ⑤ those who underwent all the following vestibular function tests: cVEMP, oVEMP, and the caloric test before treatment. The treatment protocol included administering steroids *via* intravenous and intratympanic injection and concurrent hyperbaric oxygen therapy for 10 consecutive days.

The exclusion criteria were defined as follows: central nervous system diseases, external and middle ear diseases, any cochlear and retrocochlear lesions observed on MR imaging, and hypertension or diabetes.

### Audiometry and hearing outcomes

Repeated pure-tone audiometry (PTA) was carried out before and after the 10-day treatment. PTA was measured using the clinical diagnostic audiometry system MADSEN Astera (GN Otometrics, Denmark). The average threshold was calculated based on the corresponding impaired frequency.

Hearing outcome was classified as complete recovery (hearing improvement to normal range), remarkable recovery (hearing improvement >30 dB HL), mild recovery (15 dB HL ≤ hearing improvement ≤ 30 dB HL), and no recovery (hearing improvement < 15 dB HL). Patients were tested in a standard soundproof room after removing cerumen in the external auditory canal. If no response occurred for a certain frequency, which exceeded the maximum output of the instrument (120 dB HL), 120 dB HL was used as the estimated hearing threshold.

### Vestibular evoked myogenic potential

The recording device for both cVEMP and oVEMP was the audiometry system ICS Chartr EP 200 (GN Otometrics, Denmark).

### Cervical vestibular evoked myogenic potential

A reference electrode was placed between the clavicle joints, and a ground electrode was placed between the forehead and the eyebrows. The left and right recording electrodes were placed in the middle of the left and right sternocleidomastoid muscles. The electrode impedance was <5 KΩ. The air-conducted sound was presented with 500-Hz short tone bursts (1 ms rise/fall time, 2 ms plateau time, 5 Hz stimulus frequency, and 50 times superimposition). The starting stimulus intensity was 100 dB nHL, which decreased by 5 dB nHL each time until the meaningful VEMP wave was undetectable. During the test, the subject was instructed to slightly raise his or her head by 30° to activate the sternocleidomastoid muscles.

### Ocular vestibular evoked myogenic potential

The recording parameters were similar to those in the cVEMP. The reference electrode was placed in the mandible, the ground electrode was placed between the forehead and the eyebrows, and the recording electrode was placed 1 cm below the center of the contralateral eyelid. During the test, the subject was positioned supine and stared upward (~25°-30° above the horizontal plane), trying to blink as rarely as possible to activate the inferior oblique muscles.

Recording indicators included the threshold, an initial positive peak P13(N10), a subsequent negative peak N23(P15), and P13–N23(N10–P15). The threshold value is the minimum sound stimulus intensity that elicits the typical VEMP waveform. The P13(N10) latency is the time from initiating the stimulation to the generation of the P13(N10) wave (typically 13 ms). The N23(P15) latency is the time from initiating the stimulation to the generation of the N23(P15) wave (typically 23 ms). The wave interval is the duration (ms) between the apex of the N23(P15) wave and the P13(N10) wave. The amplitude is the vertical distance (μV) from the apex of the N23(P15) wave to the apex of the P13(N10) wave. The amplitude asymmetry ratio (AR) is the ratio of the absolute value of the difference between the two sides and the sum of the two sides. An increase in AR often indicates damage to one side of the otolith organ and the superior/inferior vestibular nerve pathway. We defined the abnormal result of VEMP as (1) the absence of a meaningful waveform, (2) delayed response, whereby the threshold shift was out of the range, or (3) AR > 29%.

### Caloric test

The integrity of the external auditory canal and the tympanic membrane was checked to assess the middle ear condition before the tests were conducted. The subject was positioned supine with the head flexed at 30°. The test was performed using cold (24°) or warm (50°) air irrigation (30–60s). The nystagmus was observed for 60 s after perfusion. The canal paresis (CP) value represents the ratio of the absolute value of the difference and the total value of the left and right responses to stimulation, reflecting whether the reaction of bilateral semicircular canals was symmetrical. The directional preponderance (DP) was used to quantify the difference between the caloric responses of the two ears to judge which nystagmus direction was stronger. The abnormal caloric result was defined as an absolute value of CP% greater than 25% and/or an absolute value of DP greater than 30%.

### Statistical analysis

SPSS Statistics 26.0 was used for statistical analysis. Descriptive data were presented as mean and standard deviation values. The mean values were compared by an independent-sample *t*-test or a Kruskal–Wallis test after analyzing the normality of values using a Kolmogorov–Smirnov test (K–S test). Categorical data were expressed as number and percentage values and were compared using Pearson's χ^2^. A difference was regarded as significant if *P* < 0.05.

## Results

**Figure 1:**
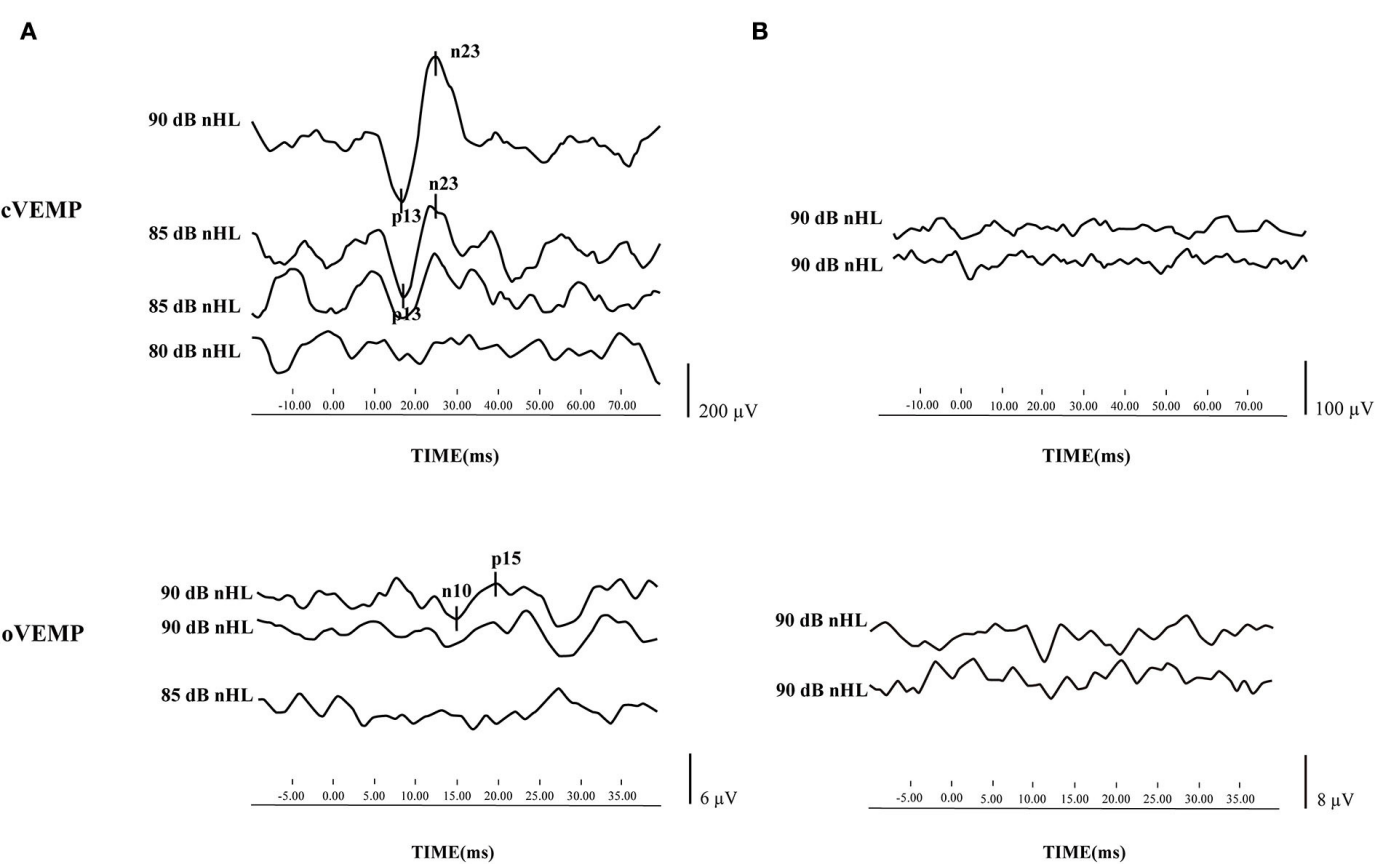
Cervical vestibular evoked myogenic potential (cVEMP) and ocular vestibular evoked myogenic potential (oVEMP) results of typical cases. **(A)** cVEMP and oVEMP were elicited from one patient with sudden sensorineural hearing loss (SSNHL) in the left ear. **(B)** cVEMP and oVEMP waveforms were absent from one patient with SSNHL in the left ear.

Before treatment, 64 patients underwent three vestibular function tests, namely, cVEMP, oVEMP, and caloric test. Only two patients had all normal results, whereas 24 patients had all abnormal results. The mean delay of treatment, defined as the period from disease onset to commencement of therapy, was 6.22 ± 5.36 days. The cVEMP and oVEMP results of typical cases are shown in Figures 1A,B.

The therapeutic efficacy was divided into two groups, namely, effective and ineffective. The effective group comprised patients with an auditory outcome showing either complete recovery, remarkable recovery, or mild recovery. In the effective group, the normal rate of cVEMP was 28.1%, and the abnormal rate was 15.6%. The normal rate of oVEMP was 17.2%, and the abnormal rate was 26.6%. In the ineffective group, the normal rate of cVEMP was 6.3%, and the abnormal rate was 50.0%. The normal rate of oVEMP was 4.7%, and the abnormal rate was 51.6%. The result of VEMP had a significant effect on the auditory outcome (*P* = 2.9 × 10^−5^, *P* = 0.008). However, the normal rate of the caloric test was 18.6%, and the abnormal rate was 37.5% in patients in the ineffective group. The caloric test results did not impact the auditory outcome in patients with UISSNHL (*P* = 0.287, Table 1).

**Table 1:** Comparison of auditory outcome in terms of vestibular function in patients with UISSNHL.

**Auditory outcome**	**cVEMP (** ***n*** **)**		**oVEMP (** ***n*** **)**		**Caloric test (** ***n*** **)**	
	**Normal**	**Abnormal**	**Normal**	**Abnormal**	**Normal**	**Abnormal**
Ineffective	4 (6.3%)	32 (50.0%)	3 (4.7%)	33 (51.6%)	12 (18.6%)	24 (37.5%)
Total effective	18 (28.1%)	10 (15.6%)	11 (17.2%)	17 (26.6%)	13 (20.3%)	15 (24.3%)
	*P* = 2.9 × 10^−5^		*P* = 0.008		*P* = 0.287	

We further investigated the contribution of vestibular function to the prognosis of patients with UISSNHL (Table 2). A better hearing improvement was observed in patients with normal cVEMP and/or oVEMP. Impaired hearing in patients with normal cVEMP improved by 33.76 ± 15.07 dB HL (*P* = 3.36 × 10^−6^), while those with abnormal cVEMP improved by 11.37 ± 17.42 dB HL. The impaired hearing in patients with normal oVEMP improved by 32.55 ± 19.56 dB HL (*P* = 0.001). However, only 15.29 ± 18.19 dB HL increased in patients with abnormal oVEMP. The caloric test results did not affect hearing improvement in patients with UISSNHL (*P* = 0.728).

**Table 2:** Relationship between vestibular function and hearing improvement.

	**cVEMP**		**Caloric test**		**oVEMP**	
	**Normal**	**Abnormal**	**Normal**	**Abnormal**	**Normal**	**Abnormal**
Hearing improvement (dB HL)	33.76 ± 15.07	11.37 ± 17.42	32.55 ± 19.56	15.29 ± 18.19	20.03 ± 19.54	18.44 ± 20.03
	*P* = 3.36 × 10^−6^		*P* = 0.001		*P* = 0.728	

Finally, we investigated the impact of VEMP results on the efficacy of patients with UISSNHL (Table 3). Of the 64 patients with UISSNHL who underwent both cVEMP and oVEMP examinations, 30 with both abnormal cVEMP and oVEMP had no response to treatment, while five with only one abnormality had no recovery from hearing impairments. Thus, the therapeutic efficacy was worse in patients with both abnormal cVEMP and oVEMP (*P* = 2.16 × 10^−4^, *P* = 0.01). Impaired hearing of patients with both normal cVEMP and oVEMP results improved by 39.24 ± 13.54 dB HL, and only one patient with both normal examinations failed to respond to treatment. Patients with either abnormal cVEMP or abnormal oVEMP results improved by 25.85 ± 17.71 dB HL. Patients with abnormal cVEMP and oVEMP results only improved by 10.90 ± 16.77 dB HL (Table 4). As a result, UISSNHL patients with both abnormal VEMP results had poorer hearing improvement.

**Table 3:** Relationship between VEMP results and patients with hearing recovery.

	**Ineffective**	**Effective**
cVEMP and oVEMP both normal	1/10 (10.0%)^*^	9/10 (90.0%)
cVEMP or oVEMP abnormal	5/16 (31.3%)^*^	11/16 (68.8%)
cVEMP and oVEMP both abnormal	30/38 (78.9%)	8/38 (21.1%)

* Compared with cVEMP and oVEMP both abnormal group, P = 2.28 × 10^−4^, P = 0.002.

**Table 4:** Relationship between VEMP result and hearing improvement.

	**Hearing improvement** **(dB HL)**	***P***
cVEMP and oVEMP both normal	39.24 ± 13.54^*^	4.75 × 10^−8^
cVEMP or oVEMP abnormal	25.85 ± 17.71^*^	
cVEMP and oVEMP both abnormal	10.90 ± 16.77	

* Compared with cVEMP and oVEMP both abnormal group, P = 2.16 × 10^−4^, P = 0.01.

## Discussion

In this study, of 10 patients with normal cVEMP and oVEMP, only one failed to respond to treatment. Therefore, patients with normal cVEMP and oVEMP had better therapeutic efficacy overall. Of the 36 patients who failed to respond to treatment, 32 had abnormal oVEMP and 33 had abnormal cVEMP. Furthermore, patients with abnormalities in both cVEMP and oVEMP had a poorer auditory outcome and an effective rate than those with only one VEMP abnormality. Those with abnormal VEMP (cVEMP and/or oVEMP) also had a relatively poorer prognosis. These results suggest that impaired function occurs in the vestibular system, most likely in the otolith organ apart from the cochlea in patients with UISSNHL. The results of cVEMP and oVEMP significantly impact the auditory outcome. One abnormality was frequently followed by another in these two tests, attributable to their high degree of consistency.

In patients with UISSNHL, the proportion of vestibular dysfunction is very high, making it crucial to conduct a vestibular evaluation in these patients (21). It has been reported that SSNHL patients with vertigo were more susceptible to a more pronounced hearing loss and had a poorer auditory outcome; hence, vertigo is likely to be a predictor of hearing improvement (3, 22, 23). In this study, 54.7% (35/64) of patients complained of vertigo, with 97.1% (34/35) of cases having vestibular dysfunction, confirming that the incidence of vestibular dysfunction is extremely high in patients with UISSNHL. However, it is worth noting that in 29 patients with UISSNHL who did not have vertigo, 96.6% (28/29) of them were accompanied by vestibular function decline, and 72.4% (21/29) of them were ineffective after treatment. Therefore, vestibular dysfunction can probably appear even in those without vertigo, and it is inappropriate to estimate whether the vestibule is involved and whether the hearing prognosis is based on symptoms of vertigo alone. Korres et al. (24) proposed that vertigo alone had no value in predicting the prognosis. You et al. (25) suggested that whether SSNHL is accompanied by vertigo or not is unrelated to the extent of inner ear damage, i.e., it is inaccurate to indicate the definite lesion site of the inner ear in patients with SSNHL.

One study in India found that 61.5% of SSNHL patients with hearing loss exceeding 90 dB HL could not elicit VEMP and sacculus damage, and the degree of hearing loss did not affect whether patients were accompanied by vertigo (26). Another study also found a better prognosis in severe and profound SSNHL patients with normal VEMP. However, the worse the vestibular function, the poorer the prognosis in patients with profound SSNHL with vertigo (24, 27). Vestibular dysfunction often indicates that patients with SSNHL have a more extensive and severe inner ear injury, while the involvement of the otolith organs suggests a relatively poorer prognosis (28). Hong et al. (29) reported that in SSNHL patients without vertigo, the abnormal rate of VEMP was higher in patients with profound high-frequency hearing loss and was positively correlated with the degree and type of hearing loss. The degree of inner ear impairment is negatively correlated with the possibility of early recovery. In this study, of 29 UISSNHL patients without vertigo, only 10 cases (34.5%) were normal in cVEMP tests, and seven cases (24.1%) were normal in oVEMP tests. This suggests that the occurrence and degree of vestibular dysfunction are not entirely related to the presence or absence of vertigo or the degree of hearing impairment.

The sacculus was more susceptible to injury than the horizontal semicircular canal in patients with SSNHL (30). This is consistent with the results of this study, which found a larger number of UISSNHL patients with abnormal cVEMP compared with those with an abnormal caloric test. Ciodaro et al. (31) investigated VEMP results in 40 patients with moderate to profound sensorineural hearing loss (MPSHL) and 30 healthy adults and found that cVEMPs were induced in 71.5% of ears in patients with MPSHL. The response rate in healthy adults was 100%, showing a high incidence of damage to the labyrinthine organs. Fujimoto et al. (4) classified SSNHL patients with vertigo based on their patterns of vestibular dysfunction and found that most of them belonged to the cochlear type, cochlear-sacculus type, and cochlear-sacculus-utriculus-semicircular canal type. Only a few patients were classified as the cochlear-utriculus type, cochlea-utriculus-horizontal semicircular canal type, and cochlea-horizontal semicircular canal type, suggesting that vestibular dysfunction in patients with SSNHL affects the vestibular organs close to the cochlea in the first place. Atrophic changes in the sacculus were observed in the vestibular organs in patients with SSNHL (5, 6, 32–34). Histopathological studies of temporal bones also reported that vestibular hair cell reduction in patients with SSNHL often occurred in the sacculus rather than in the semicircular canal (6).

The abnormal rate of the caloric test in patients with SSNHL was found to be lower than that in patients with vestibular neuritis (35). The sacculus or inferior vestibular nerve was more likely involved in SSNHL patients with vertigo, and the injury was closer to the terminal nerve, which was in a low-frequency range. Compared with the sacculus, the horizontal semicircular canal is farther away from the cochlea, making it less involved than the sacculus. However, the semicircular canal function of some patients may have been restored or compensated during the examination, and other clinical examinations are needed to evaluate the vestibular function (36). This study found that the results of the caloric test did not affect the auditory outcome of patients with UISSNHL. Of 39 patients with abnormal caloric tests, 61.5% (24/39) failed to respond to treatment. However, we noticed a considerable number of patients with abnormal caloric test outcomes in this study, and their prognosis was poor. The caloric test can evaluate the function of the bilateral horizontal semicircular canals with high sensitivity. Compared with the normal caloric test, the average hearing threshold of patients with an abnormal caloric test was higher (4). Some authors concluded that the caloric test and VEMP are predictors of early recovery of SSNHL (24). The abnormality rate of the caloric test was higher in patients with profound SSNHL. Therefore, based on these results, the cochlea, otolith organ, and semicircular canals in such patients are more likely to be affected simultaneously (24). However, the caloric test can only reflect the function of the horizontal semicircular canal, which is limited to evaluating the function of the vestibular ocular reflex (VOR) in the low-frequency range (<0.01 Hz) (37), causing poor reproducibility and intolerance in a specific group of patients.

In summary, for the first time, we combined three vestibular function tests to analyze relatively large samples and explored their influence on the auditory outcome to comprehensively predict the prognosis in patients with UISSNHL. The limitation of this study is that it included a higher proportion of older patients. They can lose the VEMP reflex, which may limit an accurate conclusion. Our results demonstrated that the prognosis of patients with abnormal cVEMP and oVEMP was poor. Generally, cVEMP and/or oVEMP are potentially valuable in predicting the outcome in patients with UISSNHL.

## Data availability statement

The raw data supporting the conclusions of this article will be made available by the authors, without undue reservation.

## Ethics statement

Written informed consent was obtained from the individual(s) for the publication of any potentially identifiable images or data included in this article.

## Author contributions

ML: data collection, writing—original draft, and funding acquisition. HW: data analysis. JC and QZ: software and validation. SL: methodology. GZ: writing—review and editing and funding acquisition. JH: visualization and funding acquisition. XC: supervision and project administration. JY: design and writing—review and editing. MD: critical revision. YJ: statistical analysis and supervision. All authors contributed to the article and approved the submitted version.

## Funding

This study was supported by the National Natural Science Foundation of China (Grant Nos. 81800903, 81970881, and 82071069).

## Conflict of interest

The authors declare that the research was conducted in the absence of any commercial or financial relationships that could be construed as a potential conflict of interest.

## Publisher's note

All claims expressed in this article are solely those of the authors and do not necessarily represent those of their affiliated organizations, or those of the publisher, the editors and the reviewers. Any product that may be evaluated in this article, or claim that may be made by its manufacturer, is not guaranteed or endorsed by the publisher.
